# Immediate versus delayed short-course ruxolitinib for acute GVHD prophylaxis after myeloablative allogeneic hematopoietic stem cell transplantation

**DOI:** 10.3389/fimmu.2026.1772778

**Published:** 2026-07-14

**Authors:** Huan Hua, Shupeng Wen, Ying Wang, Zhiyun Niu, Zhengrong Song, Ziwei Zhou, Li Sun, Zheng Xu, Jing Yuan, Zhenzhen Wang, Hang Li, Xuejun Zhang, Fuxu Wang

**Affiliations:** 1 Department of Hematology, The Second Hospital of Hebei Medical University, Shijiazhuang, China; 2 Hebei Key Laboratory of Hematology, Shijiazhuang, China; 3 Department of Histology and Embryology, Hebei Medical University, Shijiazhuang, China

**Keywords:** acute graft-versus-host disease, allogeneic hematopoietic stem cell transplantation, anti-thymocyte globulin, engraftment, haploidentical transplantation, measurable residual disease, prophylaxis, ruxolitinib

## Abstract

**Background:**

The optimal timing of ruxolitinib initiation for acute graft-versus-host disease (aGVHD) prophylaxis after myeloablative allogeneic hematopoietic stem cell transplantation (allo-HSCT) remains undefined, particularly within anti-thymocyte globulin (ATG)-based platforms. This study compared immediate versus delayed short-course ruxolitinib initiation using a concurrent two-strategy design.

**Methods:**

This single-center, retrospective, concurrent two-strategy cohort study enrolled 39 consecutive patients aged 15–65 years with hematologic malignancies who received standard “Beijing Protocol” backbone plus a 14-day short-course ruxolitinib. Patients were non-randomly assigned to immediate (day +1; n = 19) or delayed (at confirmed neutrophil engraftment; median day +13; n = 20) initiation. Haploidentical transplantation and ATG use were perfectly collinear in this cohort, complicating attribution of outcome differences. Co-primary endpoints were 100-day cumulative incidence of grade II–IV aGVHD and 2-year overall survival (OS). A 1:1 propensity score matching (PSM) analysis (12 pairs) served as a sensitivity analysis. Given the limited sample size, multivariable models were constructed as exploratory analyses and should be interpreted with caution.

**Results:**

Delayed initiation was associated with lower 100-day grade II–IV aGVHD (10.0% vs. 42.1%, P = 0.031), lower cytomegalovirus (CMV) reactivation (35.0% vs. 73.7%, P = 0.025), reduced grade ≥3 hematologic toxicity (35.0% vs. 68.4%, P = 0.043), and superior 2-year OS rate (90.0% vs. 57.9%, P = 0.012). PSM confirmed these findings (aGVHD: 8.3% vs. 41.7%, P = 0.039; OS: 91.7% vs. 58.3%, P = 0.028). In the exploratory haploidentical subgroup (n = 26), grade II–IV aGVHD was 11.8% vs. 55.6% (P = 0.028) and OS was 94.1% vs. 55.6% (P = 0.006). In exploratory multivariable models, immediate initiation was associated with higher aGVHD risk [adjusted subdistribution hazard ratio (sHR) 2.31, 95% CI 1.15–4.64, P = 0.019] and higher NRM (adjusted sHR 4.65, 95% CI 1.47–14.72, P = 0.009). Relapse risk was not significantly different (adjusted sHR 2.17, 95% CI 0.85–5.56, P = 0.095). Pre-transplant measurable residual disease (MRD) positivity was associated with inferior OS (adjusted hazard ratio [HR] 7.81, 95% CI 1.12–54.32, P = 0.038). Median follow-up was 42.1 months. Interpretation of the CMV reactivation difference is limited by the absence of donor and recipient CMV serostatus data.

**Conclusion:**

Delaying short-course ruxolitinib until neutrophil engraftment was associated with reduced aGVHD and NRM and improved survival after myeloablative allo-HSCT. No statistically significant difference in relapse risk was observed, although a clinically meaningful difference cannot be excluded. These findings warrant confirmation in a multicenter randomized trial with adequate sample size, stratified by donor type and pre-transplant MRD.

## Highlights

- Delayed ruxolitinib reduced grade II–IV acute graft-versus-host disease (aGVHD) (10.0% vs. 42.1%, P = 0.031).
- Delayed ruxolitinib was associated with superior 2-year overall survival (OS) (90.0% vs. 57.9%, P = 0.012).
- Delayed ruxolitinib substantially lowered non-relapse mortality (NRM) (5.0% vs. 31.6%, P = 0.005).
- Relapse incidence was not significantly different between groups (20.0% vs. 26.3%, P = 0.612).
- Concurrent two-strategy design mitigated temporal confounding compared with sequential cohort studies.

## 1 Introduction

Allogeneic hematopoietic stem cell transplantation (allo-HSCT) is a potentially curative therapy for a wide spectrum of hematologic malignancies, including acute myeloid leukemia (AML), acute lymphoblastic leukemia (ALL), and myelodysplastic syndromes (MDS) (1, 2). Despite continuous advances in transplant platforms and supportive care, acute graft-versus-host disease (aGVHD) remains a leading driver of non-relapse mortality (NRM), accounting for approximately 30–40% of transplant-related deaths in the first year post-transplant, and a critical barrier to successful transplantation outcomes (3). The introduction of post-transplant cyclophosphamide (PTCy)-based prophylaxis has substantially improved outcomes in modern allo-HSCT platforms; however, aGVHD and chronic GVHD (cGVHD) continue to compromise long-term survival, particularly in high-risk haploidentical transplantation (4, 5).

The Janus kinase (JAK) 1/2 inhibitor ruxolitinib has demonstrated robust efficacy in the treatment of steroid-refractory aGVHD and cGVHD in the landmark REACH2 and REACH3 trials (6, 7). The 3-year final analysis of REACH3 confirmed sustained clinical benefit and manageable safety, stimulating interest in prophylactic applications (8). Real-world multicenter experience has further corroborated these findings in both adult and pediatric populations (9). A 2025 meta-analysis highlighted extreme heterogeneity in timing (day −3 to day +45), dosage (5–10 mg twice daily), and duration (14–180 days) of prophylactic ruxolitinib across 17 studies encompassing 1,843 patients, with conflicting safety signals regarding infection risk and hematologic toxicity (10). A randomized phase II trial by Moiseev et al. demonstrated that combining ruxolitinib with PTCy was noninferior to a calcineurin inhibitor-based regimen for prevention of grade II–IV aGVHD, but did not address optimal initiation timing (11). Prolonged maintenance ruxolitinib initiated at a median of day +45 reduced moderate-severe cGVHD to 16% at 2 years (12), underscoring that the initiation window critically shapes outcomes. While this prolonged strategy primarily targets cGVHD, short-course strategies such as ours are specifically designed to cover the aGVHD pathological peak within the first 28 days post-transplant. Kröger et al. demonstrated that peri-transplant ruxolitinib initiated between day −3 and +1 reduced aGVHD in myelofibrosis patients but was associated with increased early infections (13), illustrating the therapeutic dilemma. No study has directly compared immediate versus delayed initiation within a concurrent design.

Biologically, immediate JAK inhibition on day +1 can impair dendritic cell function, cytokine-driven stem cell homing, and early antiviral interferon responses (14, 15), particularly in the context of ATG-based conditioning, which already induces profound lymphodepletion. JAK1/2 signaling is also a critical downstream pathway for stem cell factor and granulocyte colony-stimulating factor receptors; its early blockade may additionally delay hematopoietic recovery (14, 16). These concerns are especially relevant in the “Beijing Protocol”, an ATG-based myeloablative platform widely used for haploidentical transplantation in China (17–19), where severe aGVHD and CMV reactivation remain major clinical problems. The pathological peak of aGVHD occurs between days 14 and 28 post-transplant, when donor T cells have engrafted and begun to proliferate and alloreactive T-cell expansion and tissue damage reach their maximum intensity (20, 21). Delaying ruxolitinib until engraftment (median day +13) may allow therapeutic drug concentrations to be achieved during this critical window, while avoiding disruption of peri-engraftment hematopoiesis and antiviral responses. We hypothesized that delaying a standardized 14-day ruxolitinib course until confirmed neutrophil engraftment would be associated with lower incidence of grade II–IV aGVHD and improved survival.

## 2 Patients and methods

### 2.1 Study design and ethical approval

This single-center, retrospective, concurrent two-strategy cohort study was approved by the Institutional Ethics Committee of the Second Hospital of Hebei Medical University (approval number: 2019-R082) and conducted in accordance with the Declaration of Helsinki. The study was conducted and reported in accordance with the Strengthening the Reporting of Observational Studies in Epidemiology (STROBE) guidelines. Written informed consent was obtained from all patients for the transplantation procedure and the use of clinical data for retrospective research. The study was registered *post-hoc* at the Chinese Clinical Trial Registry (ChiCTR1900027971); *post-hoc* registration precludes confirmation that endpoints were pre-specified as reported and may introduce reporting bias, particularly for secondary and exploratory endpoints.

### 2.2 Study population

This retrospective study consecutively included all patients who received ruxolitinib prophylaxis and met eligibility criteria during the study period; the sample size (n = 39) was not predetermined by a formal power calculation, which increases the risk of type I and type II errors. From February to December 2019, 39 consecutive patients aged 15–65 years with hematologic malignancies were enrolled.

Inclusion criteria were: (1) histologically confirmed hematologic malignancy; (2) age 15–65 years; (3) Eastern Cooperative Oncology Group (ECOG) performance status 0–2; (4) adequate cardiac, hepatic, renal, and pulmonary function; (5) eligibility for myeloablative conditioning; (6) first allo-HSCT; (7) receipt of unmanipulated peripheral blood stem cell grafts; (8) completion of the full 14-day ruxolitinib prophylaxis regimen.

Exclusion criteria were: (1) reduced-intensity or non-myeloablative conditioning; (2) second or subsequent allo-HSCT; (3) active uncontrolled infection at transplantation; (4) history of prior solid organ transplantation; (5) pregnancy or lactation; (6) severe comorbidities precluding completion of the study protocol; (7) missing critical clinical or follow-up data (defined as absence of baseline demographic, transplant-related, or primary endpoint data).

A contemporaneous cohort of 58 patients who received standard GVHD prophylaxis without ruxolitinib during the same period was screened. These patients did not receive ruxolitinib due to physician preference, insurance issues, or patient refusal. During the study period, ruxolitinib was not covered by national health insurance in China for GVHD prophylaxis, and lower socioeconomic status is independently associated with inferior transplant outcomes; this may have introduced confounding in group assignment. After exclusion of 24 patients (12 with incomplete data, 8 lost to follow-up within the first 100 days post-transplant, 4 with severe pre-transplant comorbidities), 34 evaluable patients served as a descriptive external reference. The exclusion of eight patients from the standard cohort for loss to follow-up within the first 100 days post-transplant may have introduced survival bias. Patients lost to early follow-up are more likely to have experienced early adverse outcomes; their exclusion may render the remaining 34 evaluable standard-prophylaxis patients a selected group with better prognosis, potentially overestimating the standard cohort’s survival. No patients were lost to follow-up in the final ruxolitinib analytic cohort. Although the protocol pre-specified exclusion of patients who discontinued ruxolitinib prematurely, all 39 patients completed the full 14-day course without dose reduction or early discontinuation; therefore, this criterion did not result in any exclusions.

### 2.3 Concurrent group assignment and intervention

Throughout the study period (February 1 to December 31, 2019), two ruxolitinib initiation strategies were used concurrently with no sequential implementation. Group allocation was based on treating physician clinical judgment and patient preference; no formal criteria were used for assignment. This non-random allocation introduces selection bias, which we attempted to mitigate through propensity score matching. All patients received identical backbone GVHD prophylaxis per the “Beijing Protocol” (17, 18): cyclosporine A (1.5 mg/kg intravenously twice daily starting on day −9, tapered from day +90 to +100), mycophenolate mofetil (0.5 g orally twice daily starting on day −9, discontinued at engraftment or by day +30), and short-course methotrexate (15 mg/m² on day +1, 10 mg/m² on days +3, +6, and +11). For haploidentical transplants, rabbit ATG (2.5 mg/kg/day on days −5 to −2) was added.

Immediate Group (n = 19): Ruxolitinib 5 mg twice daily started on day +1.

Delayed Group (n = 20): Ruxolitinib started after confirmed neutrophil engraftment, defined as the first day on which ANC ≥ 0.5 × 10^9^/L was achieved and subsequently maintained for three consecutive days. In clinical practice, once the ANC reached ≥ 0.5 × 10^9^/L on a given day with a rising trend, ruxolitinib was initiated the following day, with confirmation of the three-day criterion completed retrospectively. For all 20 patients in the delayed group, the three-day engraftment criterion was ultimately met in all cases, with the first qualifying day used to define time to engraftment. The median start day was day +13 (range +12 to +20).

Both groups received an identical 14-day course: ruxolitinib 5 mg twice daily for 7 days, then 5 mg once daily for 7 days, with permanent discontinuation thereafter. Dosing was not adjusted for body weight or renal function, and pharmacokinetic monitoring was not performed; whether the 5 mg twice-daily dose achieved therapeutic drug levels in all patients cannot be confirmed. No dose reductions or interruptions occurred in any patient. This short-course strategy was designed to: (1) precisely target the pathological peak of aGVHD within the first 28 days post-transplant (20, 21); (2) align with evidence confirming efficacy of short-term JAK inhibitor prophylaxis for aGVHD risk reduction (10, 22); and (3) minimize cumulative drug exposure to reduce infection risk (14, 23).

### 2.4 Transplant procedures and supportive care

Myeloablative conditioning was disease-specific: modified BuCy (intravenous busulfan 0.8 mg/kg every 6 hours for 4 days, total 12.8 mg/kg, plus cyclophosphamide 60 mg/kg/day for 2 days) for HLA-matched sibling transplants; modified BuCy plus rabbit ATG (2.5 mg/kg/day on days −5 to −2) for haploidentical transplants; total body irradiation (TBI)-based regimens for lymphoid malignancies. G-CSF-mobilized, unmanipulated peripheral blood stem cells were used as the graft source with a target dose of ≥ 2.0 × 10^6^ CD34^+^ cells per kilogram of recipient body weight. All patients received recombinant human granulocyte colony-stimulating factor 5 μg/kg/day subcutaneously starting on day +1 until sustained neutrophil engraftment. Neutrophil engraftment was defined as ANC ≥ 0.5 × 10^9^/L for three consecutive days, with the date of engraftment recorded as the first of these three days. Platelet engraftment was defined as platelet count ≥ 20 × 10^9^/L for seven consecutive days without transfusion support, with the date of engraftment recorded as the first of these seven days. Supportive care protocols were applied identically across both groups. No letermovir prophylaxis was administered to any patient in this study; the absence of letermovir is relevant to the interpretation of CMV reactivation rates, as letermovir has been shown to significantly reduce CMV reactivation in allo-HSCT recipients.

CMV and Epstein-Barr virus (EBV) DNAemia were monitored weekly via quantitative real-time polymerase chain reaction (qPCR) in plasma with a lower limit of quantification of 100 copies/mL during the first 100 days post-transplant. Preemptive therapy with ganciclovir or foscarnet was initiated at > 1,000 copies/mL on two consecutive measurements at least 3 days apart and continued until two consecutive negative results; treatment protocols were identical across groups. This standardized preemptive strategy, rather than a prophylactic approach, contributed to the absence of CMV end-organ disease in either group.

Pre-transplant measurable residual disease (MRD) was assessed by 8-color multiparameter flow cytometry on bone marrow specimens collected within 28 days prior to conditioning, using a combination of leukemia-associated immunophenotype (LAIP) and different-from-normal (DfN) strategies. A minimum of 500,000 viable nucleated cells were acquired per specimen to ensure a reliable lower limit of detection (LLOD) of 0.01%. MRD positivity was defined as detectable disease at or above the 0.01% threshold. Acute GVHD was diagnosed and graded according to the Mount Sinai Acute GVHD International Consortium (MAGIC) criteria. Chronic GVHD was diagnosed and graded per the 2014 National Institutes of Health (NIH) Consensus Criteria. Steroid-refractory aGVHD was defined as disease progression after 3 days of treatment, or lack of clinical improvement after 7 days of treatment with methylprednisolone 1–2 mg/kg/day. Adverse events were graded per the National Cancer Institute Common Terminology Criteria for Adverse Events (CTCAE) version 5.0.

### 2.5 Endpoints

Co-primary endpoints were: (1) 100-day cumulative incidence of grade II–IV aGVHD per MAGIC criteria; (2) 2-year overall survival (OS) rate. Key secondary endpoints included: 2-year disease-free survival (DFS) rate; cumulative incidences of NRM and relapse; 100-day CMV reactivation (CMV DNA > 1,000 copies/mL in plasma on two consecutive measurements at least 3 days apart) and EBV DNAemia (EBV DNA > 1,000 copies/mL in plasma on two consecutive measurements at least 3 days apart); moderate-severe cGVHD per 2014 NIH criteria; grade ≥ 3 hematologic toxicity per CTCAE version 5.0; and time to neutrophil and platelet engraftment. The composite hematologic toxicity endpoint was defined as the occurrence of any grade ≥3 neutropenia, thrombocytopenia, or anemia within 100 days post-transplant, analyzed using crude proportions because all deaths occurred after day 100 and therefore did not act as competing events for the 100-day toxicity endpoint. Given that cytopenias in the first 100 days post-transplant reflect both transplant-related myelosuppression and potential drug-related toxicity, and that the delayed group had a drug-free interval during the peri-engraftment period (median 13 days before ruxolitinib initiation), grade ≥ 3 hematologic toxicity should be interpreted as a composite indicator of delayed or incomplete hematologic recovery rather than as a direct drug-attributable adverse event.

### 2.6 Statistical analysis

Categorical variables were compared with chi-square or Fisher’s exact test; continuous variables with the Mann-Whitney U test given the limited sample size and the non-normal distribution of most continuous variables. OS and DFS were estimated by the Kaplan-Meier method with log-rank test. Cumulative incidences of aGVHD, NRM, relapse, CMV reactivation, and EBV DNAemia were analyzed with the Fine-Gray competing risk model. For aGVHD, the competing event was death without grade II–IV aGVHD; for NRM, relapse was the competing event; for relapse, NRM was the competing event.

A landmark analysis at day +14 was performed for OS to address immortal time bias. Immortal time bias arises because patients in the delayed group must survive and remain event-free until ruxolitinib initiation to be included in the delayed group. By day +14, all patients in the delayed group had initiated ruxolitinib (range +12 to +20, median +13). The landmark analysis restricted the at-risk population to patients alive and event-free at day +14. Because no deaths occurred before day 100 (all 10 deaths: median 182 days, range 112–895 days) and no grade II–IV aGVHD events occurred before day +14, no patients were excluded by the day +14 landmark. The slight differences in P values between the landmark and primary analyses (aGVHD: P = 0.033 vs. P = 0.031; OS: P = 0.015 vs. P = 0.012) reflect minor variations in conditional probability estimation under the landmark-restricted framework and do not affect the overall conclusions. Of note, comparisons of engraftment time between groups are subject to an inherent selection bias: patients in the delayed group were required to achieve engraftment to receive ruxolitinib, whereas no such requirement existed for the immediate group. This engraftment-guaranteed selection may bias engraftment time comparisons in favor of the delayed group and should be considered when interpreting these analyses.

Propensity score matching (PSM) was performed 1:1 using nearest-neighbor matching without replacement (caliper = 0.02). The propensity score was estimated via logistic regression with group assignment as the dependent variable and age, sex, weight, pre-transplant MRD status, HLA matching, donor-recipient sex combination, and transplant calendar month as independent variables. ATG use was excluded from the propensity score model due to perfect collinearity with HLA matching (ATG was administered exclusively to haploidentical recipients). Calendar month was forced into matching to further minimize residual temporal confounding, even though the concurrent design already mitigates this bias. After matching, 12 pairs were retained with all standardized mean differences (SMD) < 0.1. The 7 unmatched immediate-group patients had a higher proportion of matched sibling donors. A sensitivity analysis using wider caliper (0.05) yielded consistent effect direction. All 10 deaths occurred after day 100 (median 182 days, range 112–895 days), justifying crude proportions for 100-day toxicity analysis.

Exploratory multivariable Fine-Gray and Cox regression models were constructed using forced-entry methodology with variables selected based on clinical relevance and P < 0.20 in univariate analysis. HLA matching and ATG use are perfectly collinear in this cohort because all haploidentical recipients received ATG while no matched sibling recipients did. For the aGVHD model (**Table 1**) and the OS model (**Table 2**), HLA matching was included as a covariate to explore its independent contribution, while ATG was excluded due to perfect collinearity with HLA matching; estimates for ruxolitinib timing in these models should be interpreted with extreme caution due to multicollinearity between ruxolitinib timing and HLA matching, which can produce unstable and misleading coefficient estimates. For the NRM and relapse models (**Table 3**), both HLA matching and ATG were excluded because the even smaller number of events (7 NRM events, 9 relapse events) precluded inclusion of highly correlated covariates. Given the limited number of events (10 deaths, 7 NRM events, 9 relapse events, 10 aGVHD events), all multivariable models are severely overfitted (events-per-variable ratio ≈ 2.3–3.0, far below the recommended minimum of 10–20). Point estimates are highly unstable, confidence intervals extremely wide, and the models cannot support causal inference. The reversal of the hazard ratio direction for ruxolitinib timing in the OS model (from univariate HR = 4.87 favoring delayed to adjusted HR = 0.267 favoring immediate) is a statistical artifact caused by multicollinearity between ruxolitinib timing and HLA matching and must not be interpreted as evidence that immediate initiation improves survival. All results from these models are exclusively hypothesis-generating and should not be independently cited as evidence of causal effects.

**Table 1:** Fine-Gray model for grade II–IV aGVHD.

Variable	Univariate sHR (95% CI)	P	Adjusted sHR (95% CI)	P
Ruxolitinib timing (Immediate vs. Delayed)	2.67 (1.42–5.01)	0.003	2.31 (1.15–4.64)	0.019
Transplant calendar month (per 1-month increase)	1.01 (0.89–1.15)	0.892	0.97 (0.85–1.11)	0.658
Pre-transplant MRD^+^ (Positive vs. Negative)	1.78 (0.96–3.30)	0.068	1.65 (0.87–3.13)	0.125
HLA matching (Haploidentical vs. Matched sibling)	1.69 (0.92–3.10)	0.089	1.58 (0.84–2.97)	0.156

sHR, subdistribution hazard ratio; CI, confidence interval; MRD, measurable residual disease; HLA, human leukocyte antigen. Competing event: death without prior grade II–IV aGVHD. ATG was excluded because of perfect collinearity with HLA matching. HLA matching was retained in the model to explore its independent contribution; however, estimates for ruxolitinib timing should be interpreted with caution due to multicollinearity between ruxolitinib timing and HLA matching. Given the limited number of events (10 events, 4 covariates; events-per-variable ratio = 2.5), this model is severely overfitted and all estimates are exclusively hypothesis-generating.

**Table 2:** Cox regression for overall survival.

Variable	Univariate HR (95% CI)	P	Adjusted HR (95% CI)	P
Ruxolitinib timing (Immediate vs. Delayed)	4.87 (1.08–21.98)	0.039	0.267 (0.048–1.491)	0.132
Calendar month (per 1-month increase)	1.03 (0.85–1.25)	0.762	1.02 (0.81–1.28)	0.874
Pre-transplant MRD^+^ (Positive vs. Negative)	4.31 (1.03–18.02)	0.045	7.81 (1.12–54.32)	0.038
HLA matching (Haploidentical vs. Matched sibling)	0.42 (0.12–1.47)	0.172	0.68 (0.15–3.12)	0.621

HR, hazard ratio; CI, confidence interval; MRD, measurable residual disease; HLA, human leukocyte antigen. Given the limited number of events (10 deaths, 4 covariates; events-per-variable ratio = 2.5), this model is severely overfitted. ATG was excluded because of perfect collinearity with HLA matching. HLA matching was retained in the model to explore its independent contribution; the reversal of the ruxolitinib timing HR direction is a statistical artifact caused by multicollinearity between ruxolitinib timing and HLA matching and must not be interpreted as evidence that immediate initiation improves survival. All estimates are exclusively hypothesis-generating.

**Table 3:** Fine-Gray models for non-relapse mortality and relapse.

Endpoint/variable	Univariate sHR (95% CI)	P	Adjusted sHR (95% CI)	P
Non-Relapse Mortality (7 events)				
Ruxolitinib timing (Immediate vs. Delayed)	5.21 (1.68–16.15)	0.005	4.65 (1.47–14.72)	0.009
Pre-transplant MRD^+^ (Positive vs. Negative)	2.08 (0.79–5.49)	0.14	1.98 (0.72–5.45)	0.186
Calendar month (per 1-month increase)	1.03 (0.85–1.25)	0.762	1.02 (0.81–1.28)	0.874
Relapse (9 events)				
Ruxolitinib timing (Immediate vs. Delayed)	1.32 (0.48–3.61)	0.595	2.17 (0.85–5.56)	0.095
Pre-transplant MRD^+^ (Positive vs. Negative)	3.76 (1.19–11.87)	0.024	3.02 (0.95–9.58)	0.061
Calendar month (per 1-month increase)	0.99 (0.82–1.19)	0.912	0.98 (0.81–1.18)	0.825

*Post-hoc* power calculation indicated 80% power to detect a 30% absolute difference in aGVHD at two-sided alpha = 0.05. This *post-hoc* estimate is driven by the observed large effect size and should not be interpreted as *a priori* evidence of adequate sample size. No formal adjustment for multiplicity was performed for the co-primary endpoints; therefore, all P values should be interpreted as nominal. The primary conclusions of this study are based on the consistency of results across both co-primary endpoints and sensitivity analyses rather than on any single P value.

Two-sided P < 0.05 was considered nominally significant. All P values are reported to three decimal places; P < 0.001 is reported as P < 0.001; P > 0.999 is reported as such from Fisher’s exact test. Analyses used SPSS 26.0 (IBM Corp., Armonk, NY, USA) and R 4.5.2 (R Foundation for Statistical Computing, Vienna, Austria) with the cmprsk package for competing risk analyses. The final follow-up date was December 31, 2022.

## 3 Results

### 3.1 Study population and baseline characteristics

Between February and December 2019, 39 consecutive patients were enrolled (median age 31 years, range 15–54). Median follow-up was 42.1 months (range 37.4–46.7) with no patients lost to follow-up in the final analytic cohort.

Baseline characteristics are shown in **Table 4**. The delayed group had significantly more haploidentical transplants (85.0% vs. 47.4%, P = 0.019) with correspondingly higher ATG use. Because haploidentical transplantation and ATG use are perfectly collinear in this cohort, their individual effects on outcomes cannot be separated. Both factors are known to influence aGVHD risk—haploidentical transplantation increases risk while ATG decreases it—and their opposing influences complicate interpretation of the full-cohort comparison. Transplant calendar months did not differ (median 6.5 vs. 7.0, P = 0.892). Pre-transplant MRD positivity was comparable (55.0% vs. 52.6%, P > 0.999). No statistically significant differences were observed in patient weight, ECOG performance status distribution, HCT-CI scores, primary diagnoses, ABO compatibility, donor-recipient sex combinations, or infused cell doses (all P > 0.05), although numerical imbalances existed for some variables given the small sample size. No patient had an ECOG performance status of 0 or ≥3.

**Table 4:** Baseline characteristics before and after propensity score matching.

Characteristic	Full cohort		P*	PSM cohort (1:1, 12 pairs)		SMD
	Delayed (n = 20)	Immediate (n = 19)		Delayed (n = 12)	Immediate (n = 12)	
Demographics						
Age, years, median (range)	29 (15–54)	34 (17–52)	0.168	31 (15–54)	32 (17–52)	0.088
Male sex, n (%)	10 (50.0)	11 (57.9)	0.751	6 (50.0)	6 (50.0)	0
Weight, kg, median (range)	66 (48–88)	63 (45–85)	0.279	65 (48–82)	64 (45–85)	0.056
ECOG Performance Status, n (%)			0.480			0.065
ECOG 1	16 (80.0)	13 (68.4)		10 (83.3)	9 (75.0)	
ECOG 2	4 (20.0)	6 (31.6)		2 (16.7)	3 (25.0)	
HCT-CI, n (%)			0.713			0.042
HCT-CI 0	1 (5.0)	2 (10.5)		1 (8.3)	1 (8.3)	
HCT-CI 1–2	18 (90.0)	16 (84.2)		10 (83.3)	10 (83.3)	
HCT-CI ≥ 3	1 (5.0)	1 (5.3)		1 (8.3)	1 (8.3)	
Disease characteristics						
AML, n (%)	10 (50.0)	7 (36.8)		6 (50.0)	5 (41.7)	
ALL, n (%)	7 (35.0)	8 (42.1)		4 (33.3)	5 (41.7)	
MDS, n (%)	2 (10.0)	1 (5.3)		1 (8.3)	1 (8.3)	
Other, n (%)a	1 (5.0)	3 (15.8)	0.652b	1 (8.3)	1 (8.3)	0.078b
Pre-transplant MRD^+^, n (%)	11 (55.0)	10 (52.6)	> 0.999	6 (50.0)	6 (50.0)	0
Transplant characteristics						
Haploidentical donor, n (%)	17 (85.0)	9 (47.4)	0.019	9 (75.0)	9 (75.0)	0
ATG use, n (%)	17 (85.0)	9 (47.4)	0.019	9 (75.0)	9 (75.0)	0
Donor-recipient sex mismatch, n (%)	10 (50.0)	10 (52.6)	0.751	6 (50.0)	6 (50.0)	0
ABO incompatible, n (%)	10 (50.0)	9 (47.4)	> 0.999	6 (50.0)	6 (50.0)	0
Calendar month, median (range)	6.5 (2–12)	7.0 (2–12)	0.892	6.0 (2–12)	7.0 (2–12)	0.045
MNC, × 10^8^ cells/kg, median (range)	11.15 (6.72–17.30)	10.68 (6.19–18.23)	0.698	11.02 (6.72–17.30)	10.87 (6.19–18.23)	0.032
CD34^+^, × 10^6^ cells/kg, median (range)	4.54 (2.55–10.20)	4.39 (2.98–8.00)	0.923	4.48 (2.55–10.20)	4.42 (2.98–8.00)	0.021

ECOG, Eastern Cooperative Oncology Group; HCT-CI, Hematopoietic Cell Transplantation-Comorbidity Index; AML, acute myeloid leukemia; ALL, acute lymphoblastic leukemia; MDS, myelodysplastic syndromes; MRD, measurable residual disease; ATG, anti-thymocyte globulin; MNC, mononuclear cells; CD34+, Hematopoietic stem cells expressing the CD34 surface marker; SMD, standardized mean difference; HLA, human leukocyte antigen. No patient had an ECOG performance status of 0 or ≥3.

^a^Other includes chronic myeloid leukemia (delayed n = 0, immediate n = 2) and non-Hodgkin lymphoma (delayed n = 1, immediate n = 1). ^b^P value for overall diagnosis distribution (chi-square test). ^c^P values correspond to the comparison between delayed and immediate groups in the full cohort (Mann-Whitney U test for continuous variables; Fisher’s exact test for categorical variables). SMD values are reported for the PSM cohort.

**Figure 1:**
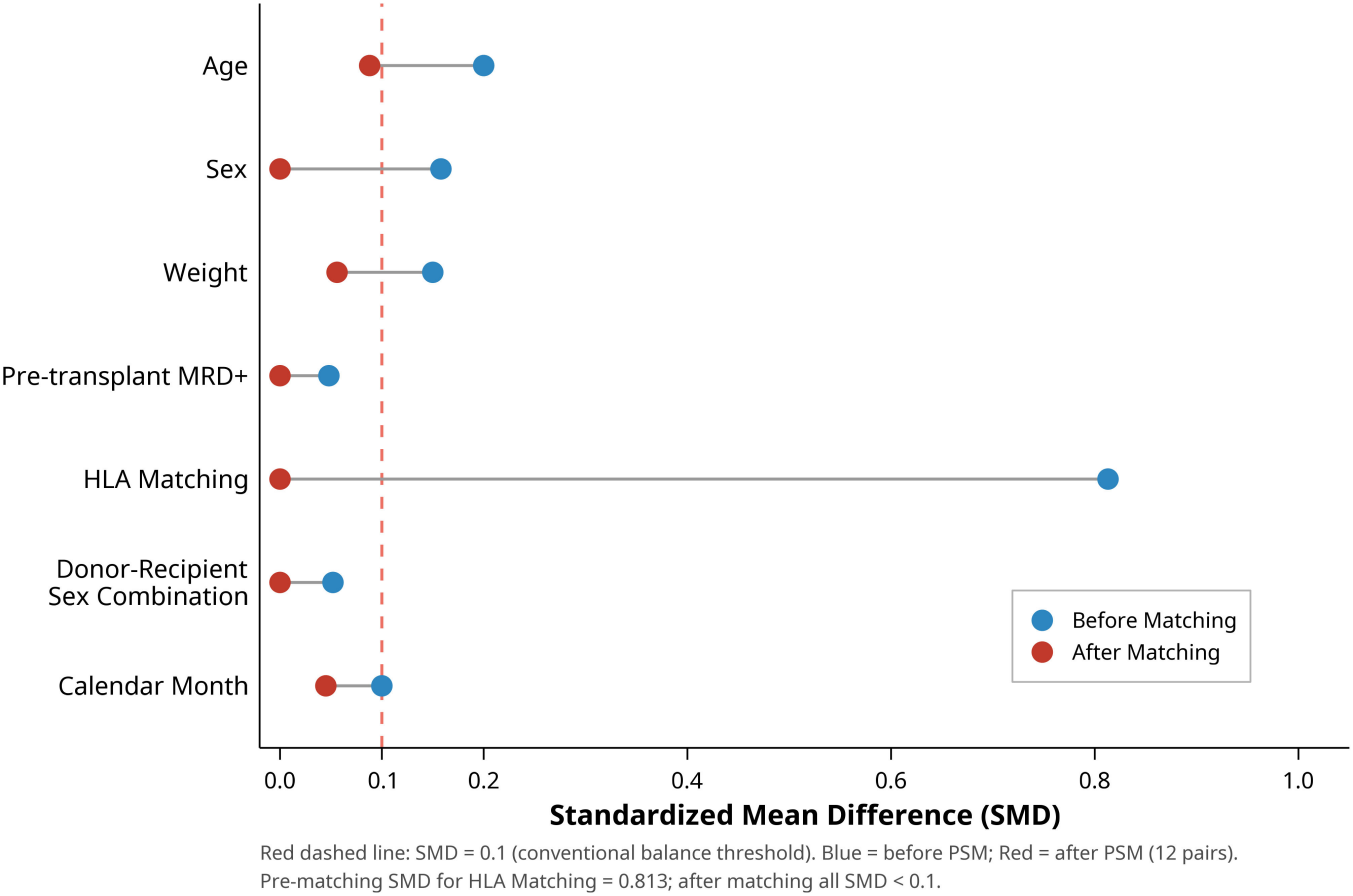
Love plot of standardized mean differences before and after propensity score matching. Detailed description provided at the end of the manuscript.

After 1:1 PSM (12 pairs), all covariates achieved SMD < 0.1 (**Figure 1**, **Table 4**). HLA matching and ATG use—the variables with the largest baseline imbalance (SMD = 0.813)—were fully balanced after matching (SMD = 0.000).

### 3.2 Engraftment and toxicity

**Figure 2:**
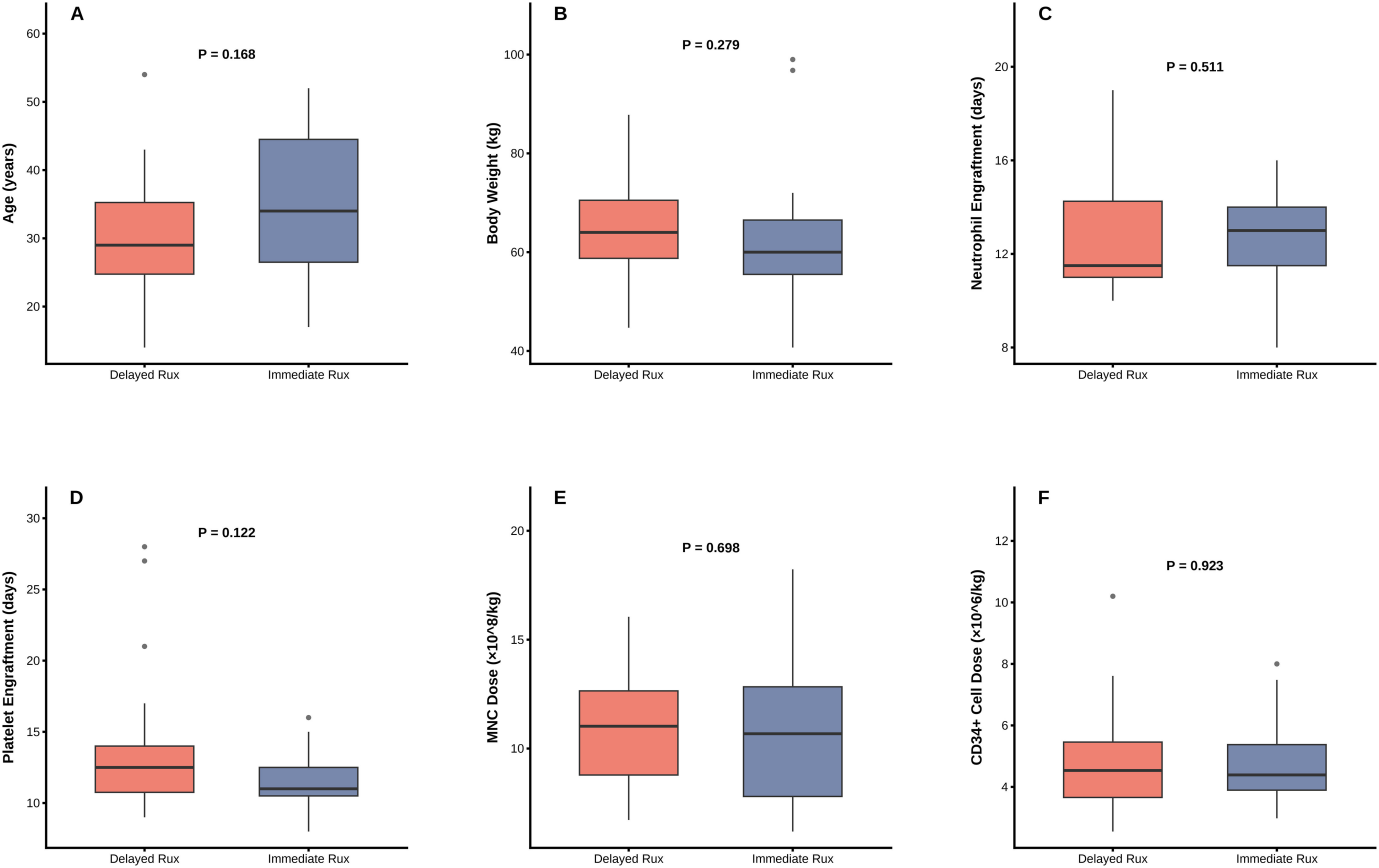
Comparative analysis of baseline and engraftment parameters between delayed and immediate ruxolitinib groups. **(A)** age distribution, **(B)** body weight, **(C)** time to neutrophil engraftment, **(D)** time to platelet engraftment, **(E)** MNC dose, and **(F)** CD34^+^ cell dose between the two groups, with Wilcoxon rank-sum test P values annotated.

All 39 patients achieved sustained engraftment. Median neutrophil engraftment (first day of ANC ≥ 0.5 × 10^9^/L subsequently sustained for three days) was 11.5 days in the delayed group and 13 days in the immediate group (P = 0.511). Although this difference was not statistically significant, the direction of the difference (faster neutrophil recovery in the delayed group) is consistent with the biological rationale that early JAK inhibition may impair hematopoietic stem cell engraftment and early myeloid recovery. Median platelet engraftment was 12.5 days in the delayed group and 11 days in the immediate group (P = 0.122) (**Figure 2**). Of note, in the delayed group, the median platelet engraftment (day +12.5) preceded the median ruxolitinib initiation (day +13), indicating that platelet recovery in the delayed group was largely accomplished before ruxolitinib exposure and was therefore unaffected by the drug. The slightly shorter median platelet engraftment time in the immediate group may partly reflect the higher proportion of matched sibling donors in that group (52.6% vs. 15.0%), as HLA-matched transplants generally exhibit more rapid hematopoietic recovery compared with haploidentical transplants. Furthermore, comparisons of engraftment time between groups are subject to selection bias: patients in the delayed group were required to achieve engraftment to receive ruxolitinib, whereas no such requirement existed for the immediate group, potentially favoring the delayed group in engraftment time comparisons.

**Figure 3:**
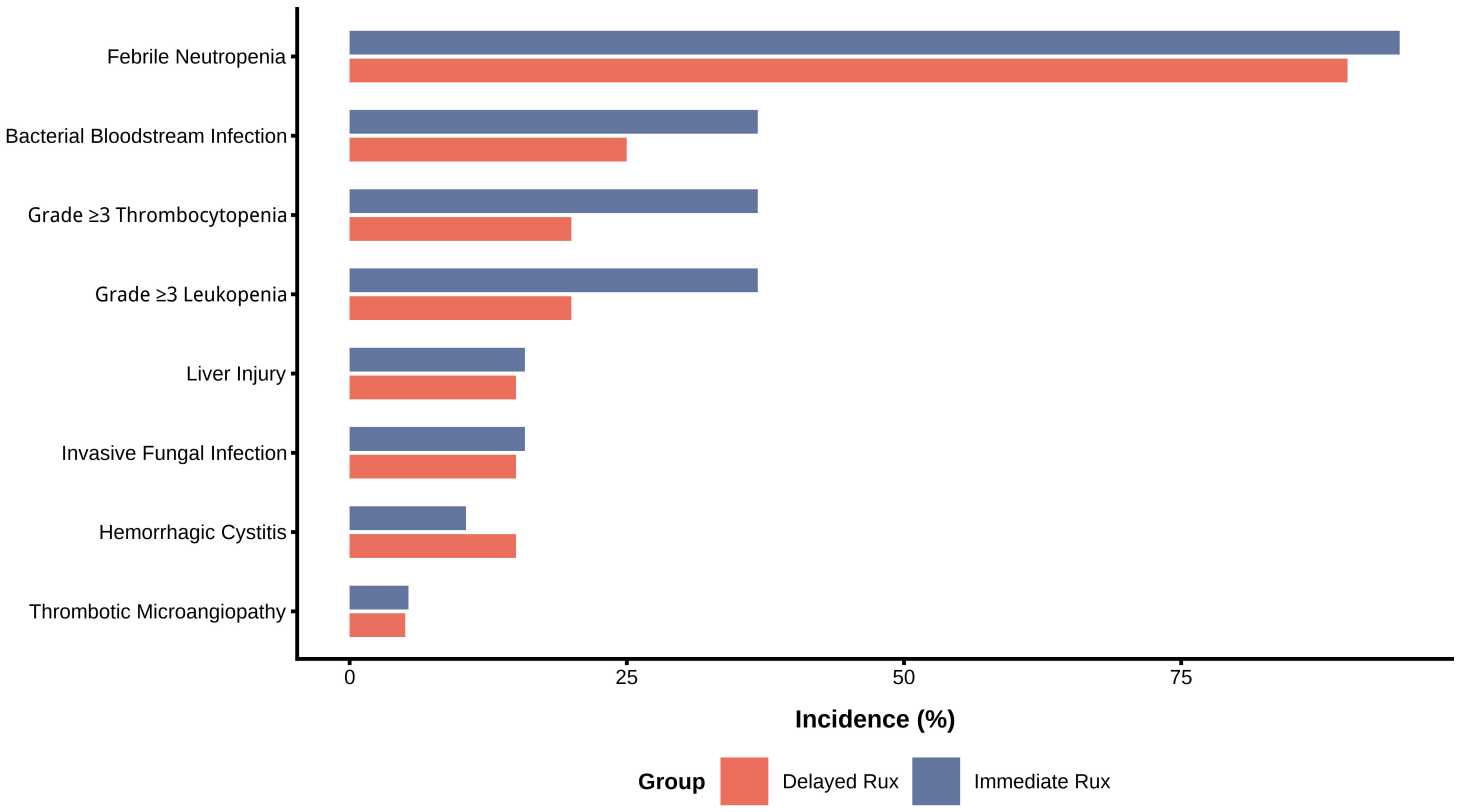
Incidence of grade ≥3 treatment-emergent adverse events in the two groups.

The delayed group had significantly lower grade ≥ 3 hematologic toxicity (35.0% vs. 68.4%, P = 0.043; **Figure 3**, **Table 5**). Given the drug-free interval during the peri-engraftment period in the delayed group (median 13 days from transplant to ruxolitinib initiation), the observed difference in grade ≥3 hematologic toxicity likely reflects more rapid hematopoietic recovery rather than a direct ruxolitinib-related toxic effect. Grade ≥ 3 neutropenia occurred in 20.0% vs. 47.4% (P = 0.089), thrombocytopenia in 15.0% vs. 36.8% (P = 0.142), and anemia in 10.0% vs. 21.1% (P = 0.412). Febrile neutropenia occurred in 90.0% vs. 94.7% (P > 0.999), without infection-related deaths during neutropenia in either group. In the matched cohort, grade ≥ 3 hematologic toxicity was 33.3% vs. 66.7% (P = 0.041).

**Table 5:** Post-transplant complications.

Complication	Full cohort		P	Haploidentical subgroup		P
	Delayed (n = 20)	Immediate (n = 19)		Delayed (n = 17)	Immediate (n = 9)	
Any grade aGVHD, %	25	42.1	0.320a	23.5	55.6	0.198a
Grade II–IV aGVHD, %	10	42.1	0.031a	11.8	55.6	0.028a
Steroid-refractory aGVHD, nb	0	2	—c	0	2	—c
Any grade cGVHD, %	30	21.1	0.716a	35.3	11.1	0.375a
Moderate-severe cGVHD, %	15	15.8	> 0.999a	17.6	11.1	> 0.999a
CMV reactivation, %	35	73.7	0.025a	35.3	77.8	0.047a
EBV DNAemia, %	20	31.6	0.480a	17.6	33.3	0.631a
Grade ≥ 3 hematologic toxicity, %	35	68.4	0.043d	35.3	66.7	0.217d
Febrile neutropenia, %	90	94.7	> 0.999d	88.2	100	0.537d
Invasive fungal infection, %	15	15.8	> 0.999d	11.8	22.2	0.606d
Bacterial bloodstream infection, %	25	36.8	0.507d	23.5	44.4	0.404d

aGVHD, acute graft-versus-host disease; cGVHD, chronic graft-versus-host disease; CMV, cytomegalovirus; EBV, Epstein-Barr virus. cGVHD cumulative incidences were estimated at 2 years post-transplant.

^a^P values from Gray’s test for cumulative incidences. ^b^Both steroid-refractory aGVHD events occurred exclusively in the immediate group and in haploidentical transplant recipients. ^c^P value not reported because only two steroid-refractory aGVHD events occurred; statistical testing with two events is uninformative. ^d^P values from Fisher’s exact test for crude proportions (all deaths occurred after day 100).

### 3.3 Acute GVHD

**Figure 4:**
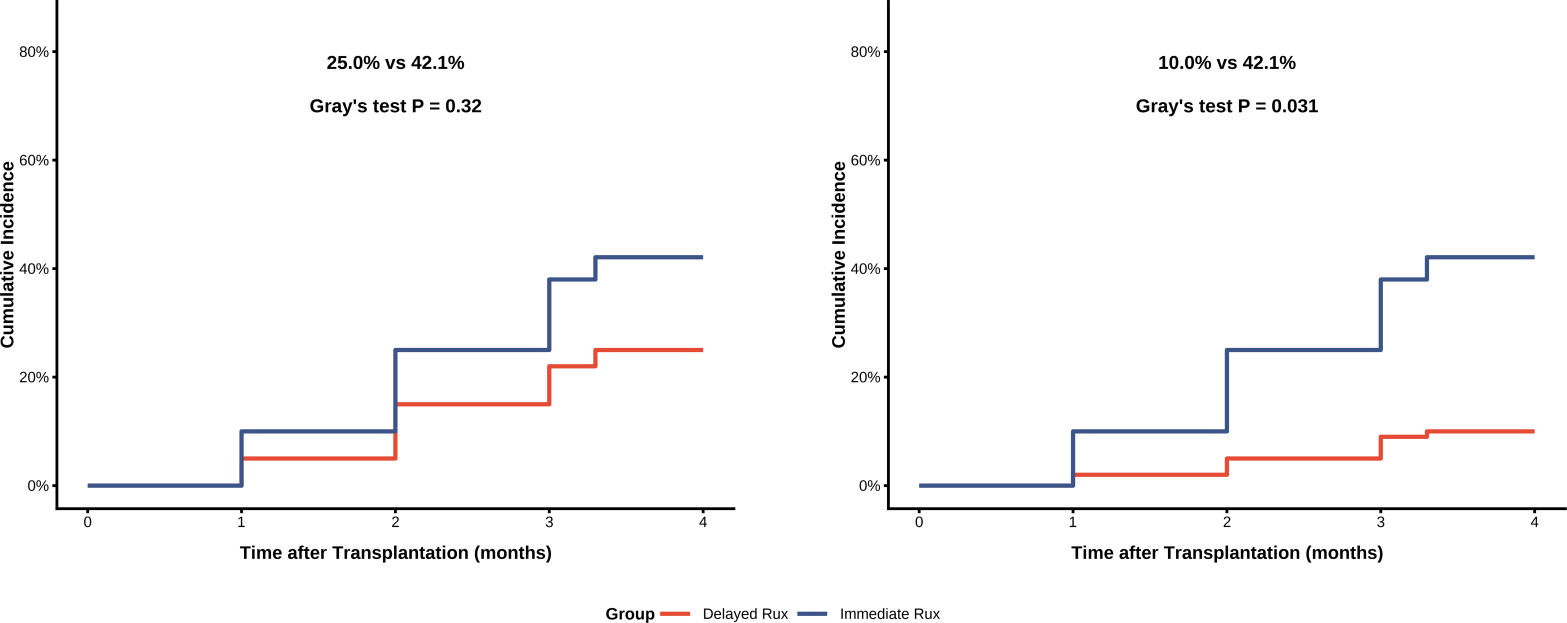
Cumulative incidence of acute GVHD by ruxolitinib initiation timing. **(A)** Cumulative incidence of any grade aGVHD; **(B)** Cumulative incidence of grade II–IV aGVHD, with Gray’s test P values annotated.

The 100-day cumulative incidence of grade II–IV aGVHD was significantly lower in the delayed group (10.0% vs. 42.1%, Gray’s test P = 0.031; **Figure 4B**, **Table 5**). Any-grade aGVHD cumulative incidence did not differ significantly (25.0% vs. 42.1%, Gray’s test P = 0.320; **Figure 4A**). Both cases of steroid-refractory aGVHD (both grade IV gastrointestinal disease) occurred exclusively in the immediate group and both occurred in haploidentical transplant recipients. With only two steroid-refractory events, formal statistical testing is uninformative; these events are therefore described qualitatively. The median onset of aGVHD was day +41 in the delayed group versus day +28 in the immediate group (P = 0.073). Although this 13-day difference did not reach statistical significance, a later onset of aGVHD may be clinically relevant, as it allows more time for hematologic recovery and may be associated with a reduced risk of infectious complications during subsequent corticosteroid therapy.

In the PSM cohort, grade II–IV aGVHD was 8.3% vs. 41.7% (P = 0.039) and 2-year OS rate was 91.7% vs. 58.3% (P = 0.028). Secondary endpoints in the matched cohort showed directionally consistent results: 2-year NRM was 8.3% vs. 33.3%, 2-year DFS rate was 75.0% vs. 41.7%, CMV reactivation was 33.3% vs. 66.7%, and grade ≥ 3 hematologic toxicity was 33.3% vs. 66.7%; relapse and cGVHD did not differ substantially between matched groups.

In an exploratory Fine-Gray model (**Table 1**), immediate initiation was associated with higher aGVHD risk (adjusted sHR 2.31, 95% CI 1.15–4.64, P = 0.019), while calendar month was not significant (adjusted sHR 0.97, 95% CI 0.85–1.11, P = 0.658). The non-significant calendar month effect confirms that the aGVHD difference between groups is not driven by temporal trends. Readers should note that given the limited number of events (10 events, 4 covariates; events-per-variable ratio = 2.5), this model is severely overfitted and all estimates are exclusively hypothesis-generating. .

### 3.4 Haploidentical high-risk subgroup analysis

**Figure 5:**
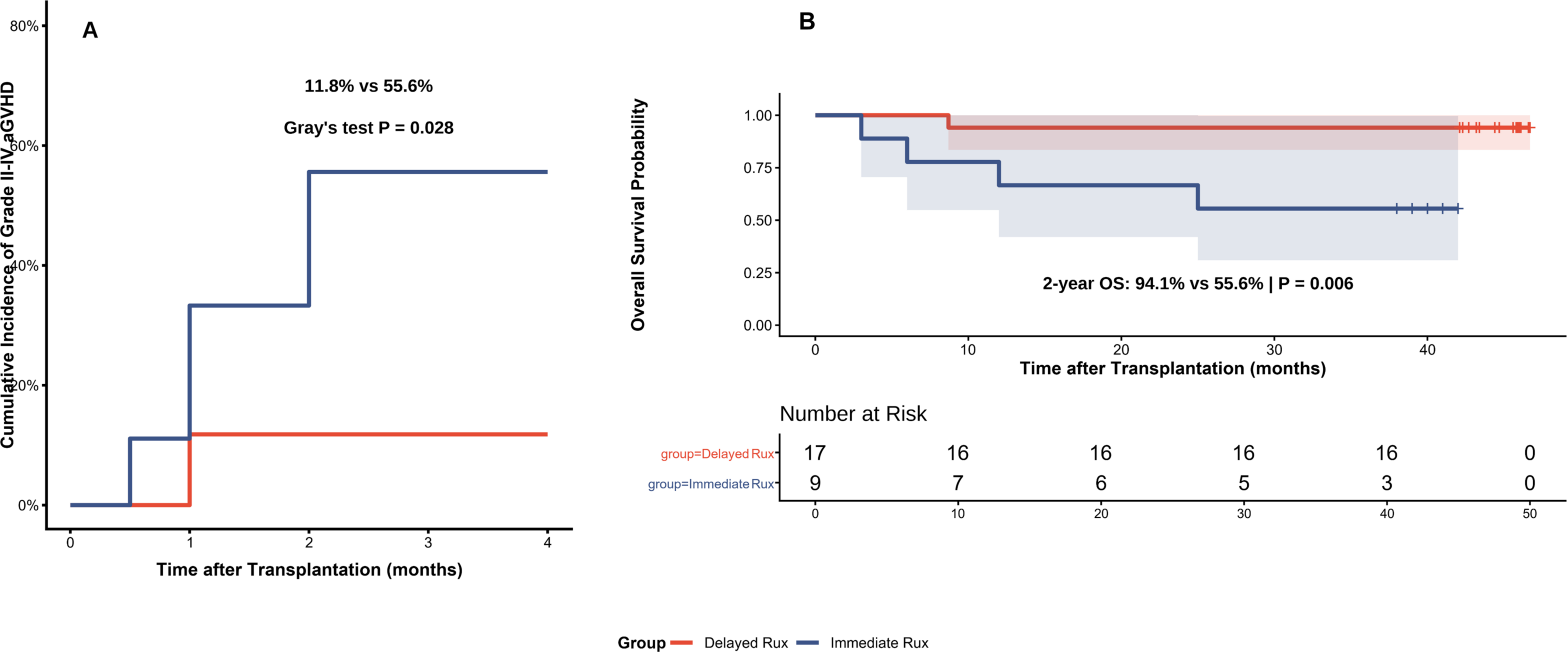
Core endpoints in the HFD high-risk subgroup. **(A)** Cumulative incidence of grade II–IV aGVHD in the HFD subgroup; **(B)** Kaplan-Meier curves for overall survival in the HFD subgroup, with log-rank P value and 2-year survival rates annotated.

In the exploratory haploidentical subgroup (n = 26), grade II–IV aGVHD was 11.8% in the delayed group versus 55.6% in the immediate group (Gray’s test P = 0.028; **Figure 5A**, **Table 5**), and 2-year OS rate was 94.1% versus 55.6% (log-rank P = 0.006; **Figure 5B**). No formal interaction test was performed due to limited sample size; even if conducted, the test would have insufficient power to detect a meaningful interaction between donor type and ruxolitinib timing. In the absence of a statistically significant interaction test, the apparently larger benefit in haploidentical recipients remains a hypothesis-generating observation. In the full cohort, the delayed group had a higher proportion of haploidentical transplants (85.0% vs. 47.4%) and correspondingly higher ATG use. Because haploidentical transplantation and ATG are perfectly collinear, their individual contributions to the observed outcome differences cannot be isolated; the higher ATG use in the delayed group may have contributed to the lower aGVHD rate in addition to the effect of ruxolitinib timing. This subgroup analysis is exploratory and requires confirmation in larger cohorts designed to disentangle donor type from ATG exposure.

### 3.5 Viral reactivation, cGVHD, relapse, and survival

**Figure 6:**
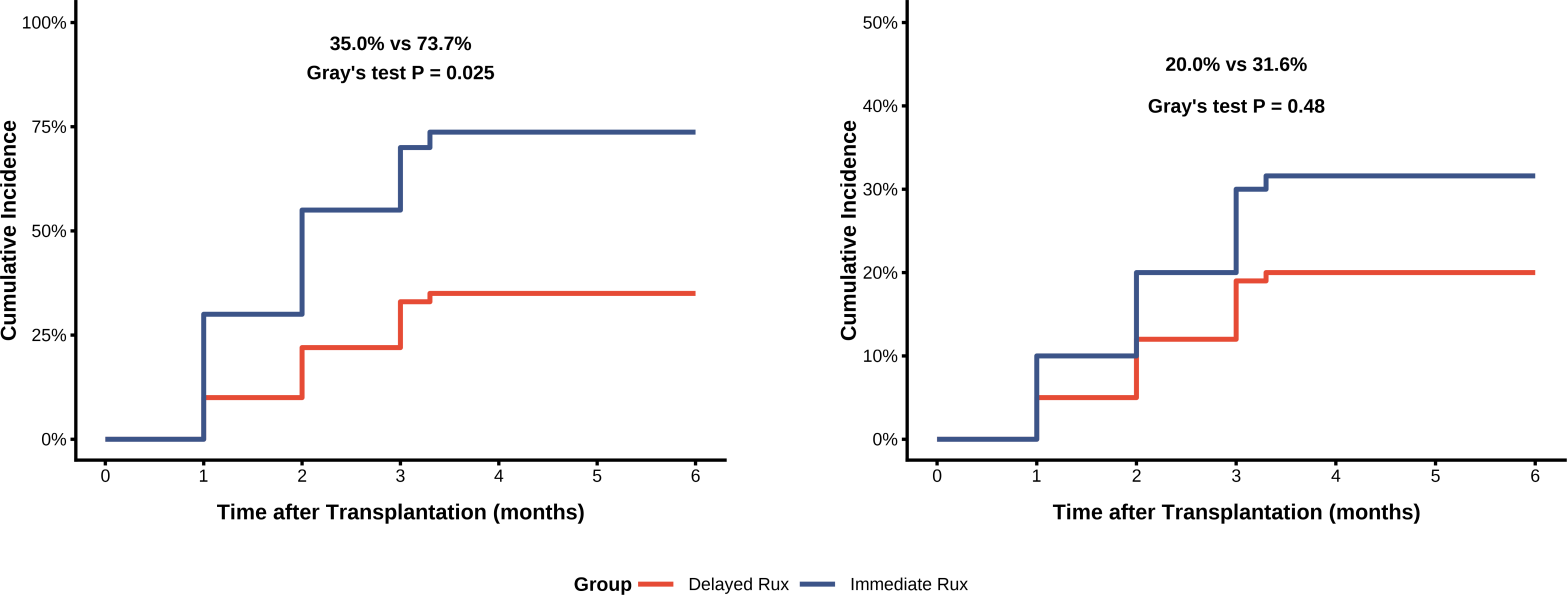
Viral reactivation cumulative incidence by treatment group. **(A)** Cumulative incidence of CMV reactivation; **(B)** Cumulative incidence of EBV reactivation, with Gray’s test P values annotated.

The 100-day cumulative incidence of CMV reactivation was significantly lower in the delayed group (35.0% vs. 73.7%, Gray’s test P = 0.025; **Figure 6A**, **Table 5**). Interpretation of this difference is critically limited by the absence of donor and recipient CMV serostatus data, which are the most important determinants of post-transplant CMV reactivation. EBV DNAemia cumulative incidence was 20.0% vs. 31.6% (Gray’s test P = 0.480; **Figure 6B**). No cases of EBV-associated post-transplant lymphoproliferative disorder occurred in either group during follow-up. Two-year cumulative incidence of cGVHD did not differ significantly between groups (any grade: 30.0% vs. 21.1%, Gray’s test P = 0.716; moderate-severe: 15.0% vs. 15.8%, Gray’s test P > 0.999). The clinical phenotypes of cGVHD differed descriptively: the delayed group exhibited predominantly mild gastrointestinal involvement (66.7% of cases), whereas the immediate group showed more diverse multi-organ involvement including fascial, joint, and hepatic disease. Differences in cGVHD organ involvement cannot be formally tested given the small number of events (6 cases in the delayed group, 4 in the immediate group) and are purely descriptive. All cGVHD cases in the delayed group occurred in haploidentical recipients, while three of four cGVHD cases in the immediate group occurred in matched sibling recipients.

**Figure 7:**
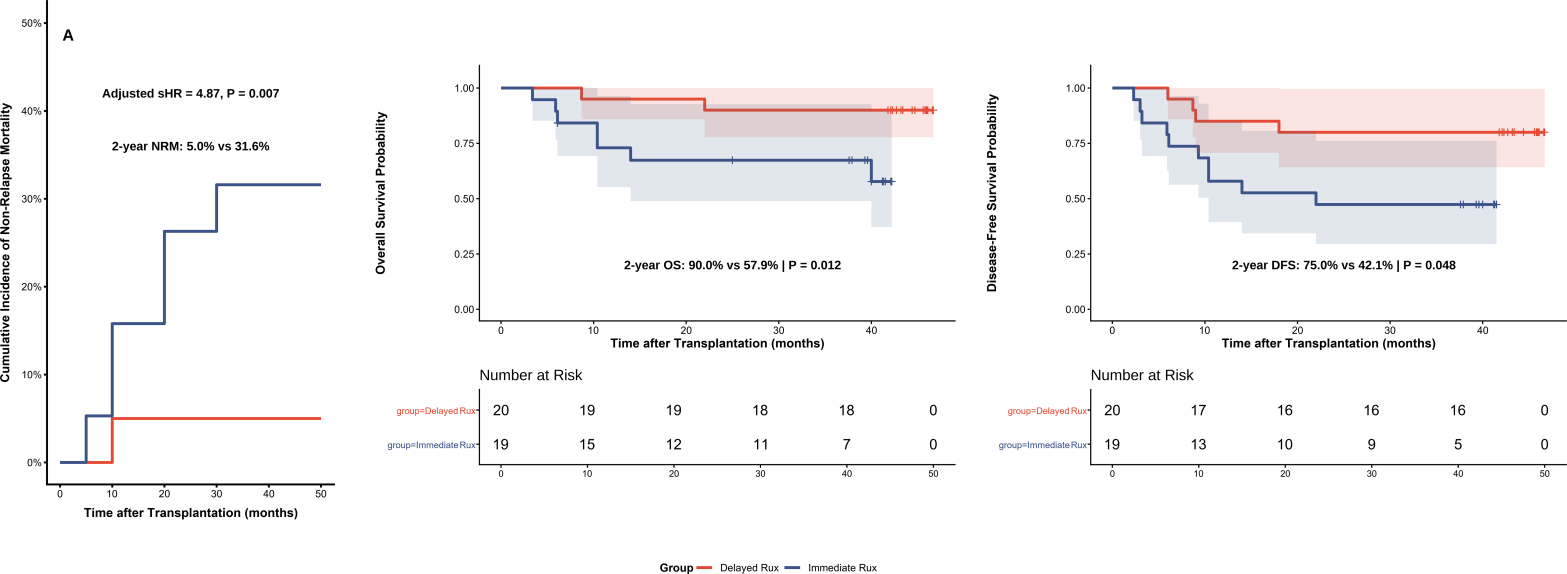
Long-term survival and competing risk outcomes. **(A)** Cumulative incidence of non-relapse mortality (NRM); **(B)** Kaplan-Meier curves for overall survival (OS); **(C)** Kaplan-Meier curves for disease-free survival (DFS), with log-rank P values and 2-year event rates annotated.

Cumulative incidence of relapse was 20.0% vs. 26.3% (Gray’s test P = 0.612). Cumulative incidence of NRM was markedly reduced in the delayed group (5.0% vs. 31.6%, Gray’s test P = 0.005; **Figure 7A**), translating to superior 2-year OS rate (90.0% vs. 57.9%, log-rank P = 0.012; **Figure 7B**) and 2-year DFS rate (75.0% vs. 42.1%, log-rank P = 0.048; **Figure 7C**). The improved DFS in the delayed group was driven primarily by the reduction in NRM rather than by differences in relapse. Among the 9 patients who experienced disease relapse (4 delayed, 5 immediate), 3 died of relapsed disease (1 delayed at 14 months, 2 immediate at 6 and 10 months); the remaining 6 were alive at last follow-up with salvage therapy.

A total of 10 deaths occurred: 7 due to NRM and 3 relapse-related deaths. Of the 10 deaths, 5 occurred in haploidentical recipients (1 delayed, 4 immediate) and 5 in matched sibling recipients (1 delayed, 4 immediate). The sole NRM event in the delayed group was a late-onset pulmonary infection at 8 months post-transplant in a matched sibling recipient. NRM events in the immediate group (n = 6 total) comprised severe refractory gastrointestinal GVHD (n = 2, both haploidentical recipients), cerebral hemorrhage related to prolonged thrombocytopenia and corticosteroid use (n = 1, haploidentical recipient), and severe bacterial or fungal infections (n = 3; 1 haploidentical, 2 matched sibling). Both patients with steroid-refractory aGVHD died of refractory GVHD, accounting for two of the six NRM events in the immediate group (33.3%) and 28.6% of all NRM events across both groups.

### 3.6 External PSM-matched comparison with standard prophylaxis

All 20 patients in the delayed ruxolitinib group were matched 1:1 to 20 patients from the standard prophylaxis cohort using the same covariates as the internal PSM; all SMD values after matching were < 0.1. Fourteen patients from the standard prophylaxis cohort were not matched due to insufficient covariate overlap, suggesting that the matched standard cohort may not be fully representative of the entire standard prophylaxis population. This analysis is subject to significant selection bias because patients did not receive ruxolitinib due to physician preference, insurance issues, or personal refusal. Furthermore, 8 patients were excluded from the standard cohort due to loss to follow-up within the first 100 days post-transplant (versus zero in the ruxolitinib cohort); their exclusion may have rendered the remaining standard-prophylaxis patients a selected group with better prognosis. Results are presented as descriptive point estimates without formal statistical inference, as the biases preclude meaningful hypothesis testing.

**Figure 8:**
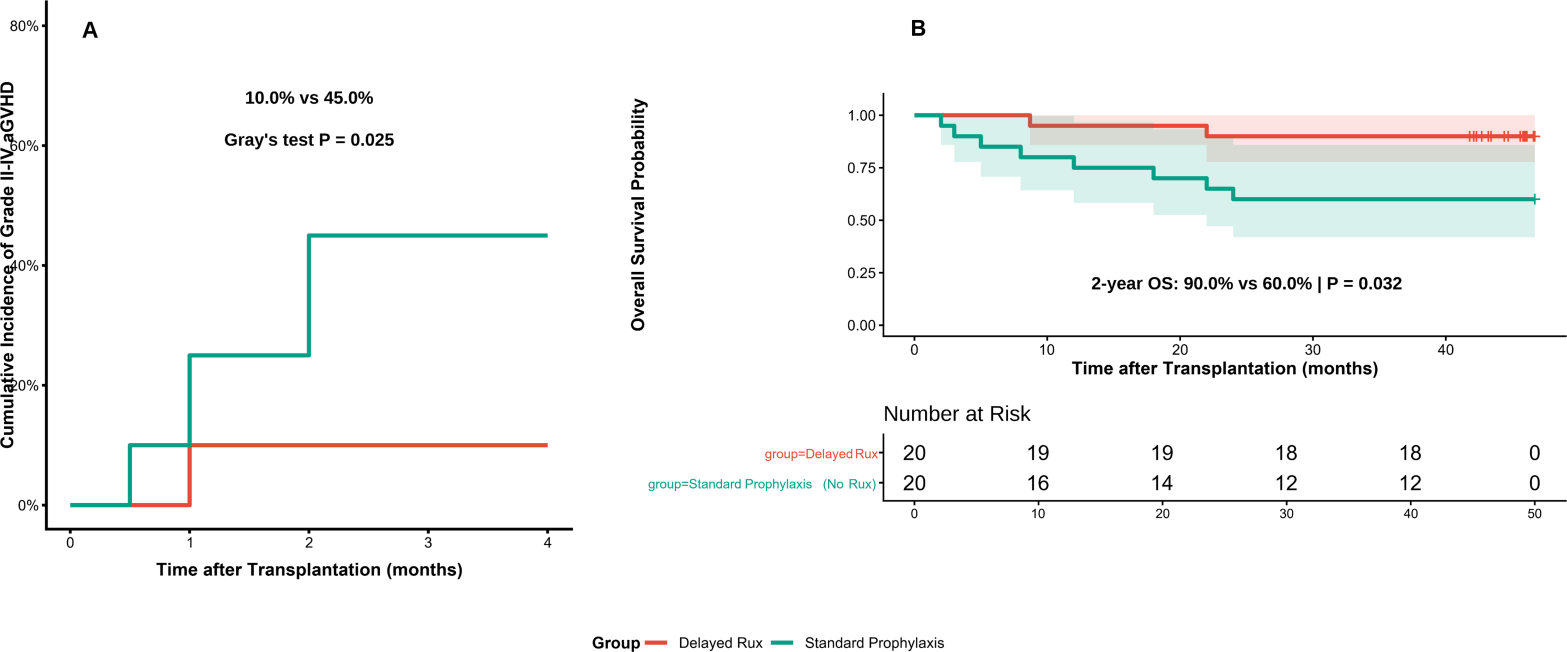
Delayed ruxolitinib vs standard prophylaxis cohort (PSM-Matched). **(A)** Cumulative incidence of grade II–IV aGVHD; **(B)** Kaplan-Meier curves for overall survival, with Gray’s test and log-rank P values annotated.

In this descriptive comparison, the 100-day cumulative incidence of grade II–IV aGVHD was 10.0% in the delayed ruxolitinib group and 45.0% in the standard prophylaxis group (**Figure 8A**). The 2-year OS rate was 90.0% and 60.0%, respectively (**Figure 8B**). The 2-year DFS rate was 75.0% and 50.0%, 2-year NRM was 5.0% and 30.0%, 100-day CMV reactivation was 35.0% and 60.0%, and 2-year relapse was 20.0% and 25.0% (**Table 6**). The 2-year OS rate of the delayed group was 90.0% in both the internal full cohort and this external comparison, suggesting consistency of the observed effect magnitude. However, given the substantial biases inherent in this comparison, this numerical concordance should not be overinterpreted as evidence of absence of residual confounding. This comparison is presented solely for descriptive completeness and cannot support clinical inference. .

**Table 6:** Descriptive comparison of delayed ruxolitinib versus standard prophylaxis (PSM-matched).

Endpoint	Delayed ruxolitinib (n = 20)	Standard prophylaxis (n = 20)
100-day Grade II–IV aGVHD, %	10	45
2-year Overall Survival rate, %	90	60
2-year Disease-Free Survival rate, %	75	50
2-year Non-Relapse Mortality, %	5	30
2-year Relapse, %	20	25
100-day CMV Reactivation, %	35	60
100-day EBV DNAemia, %	20	35

aGVHD, acute graft-versus-host disease; CMV, cytomegalovirus; EBV, Epstein-Barr virus. Point estimates only; no P values are presented because this external comparison is subject to significant selection bias. Data are presented for descriptive completeness only and cannot support clinical inference.

### 3.7 Exploratory prognostic factor analyses

Given the limited number of events (10 deaths, 7 NRM events, 9 relapse events), the following multivariable models were used to explore potential associations. All results from this analysis should be interpreted with caution and are exclusively hypothesis-generating; point estimates are unstable and confidence intervals are extremely wide.

**Figure 9:**
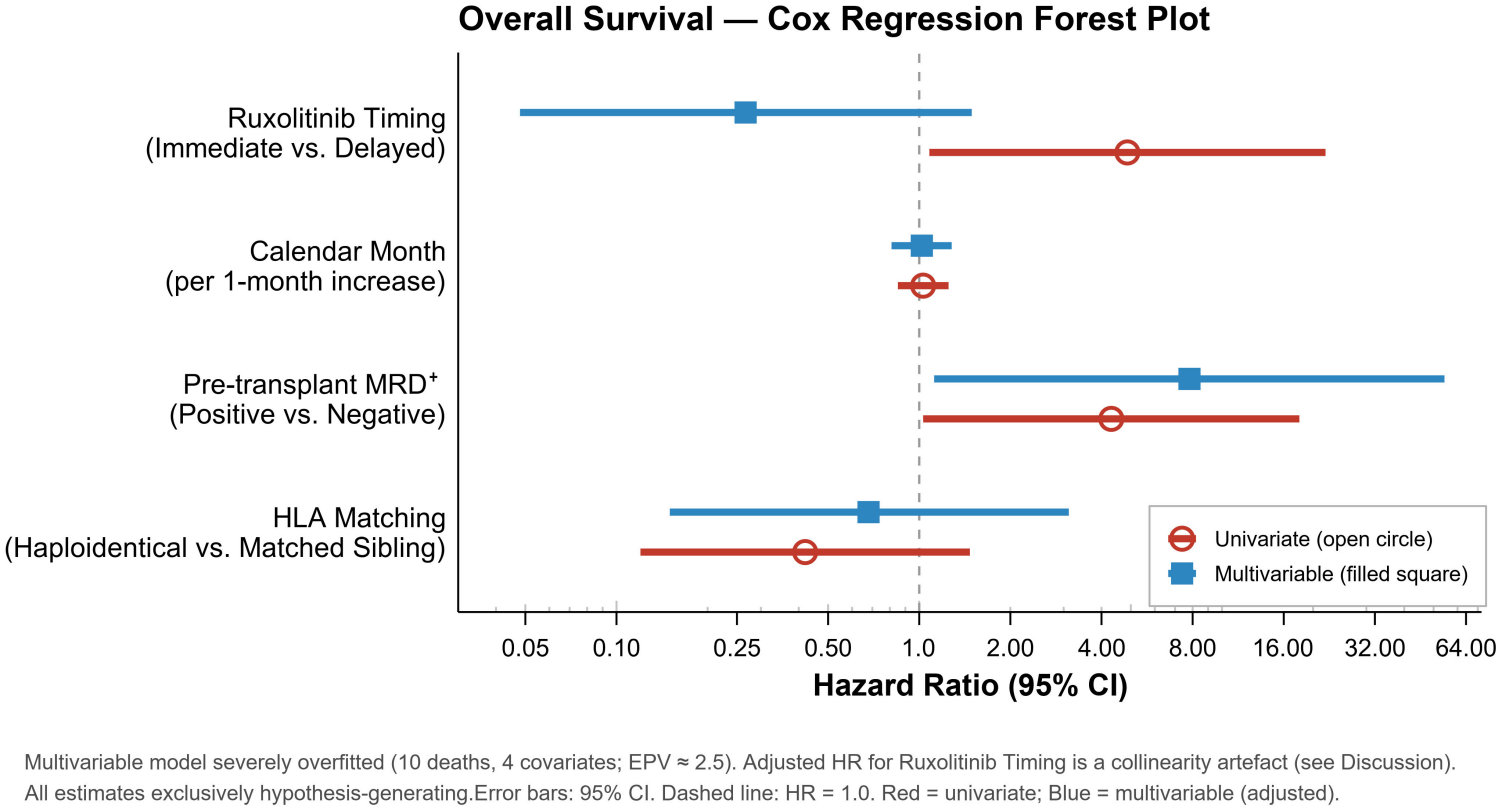
Forest plot of Cox regression for overall survival. Detailed description provided at the end of the manuscript.

In the exploratory Cox model for OS (**Table 2**, **Figure 9**), pre-transplant MRD positivity was most strongly associated with inferior OS (adjusted HR 7.81, 95% CI 1.12–54.32, P = 0.038). The extremely wide confidence interval reflects the small number of events (10 deaths) and indicates considerable uncertainty in the estimate. The effect of ruxolitinib timing reversed direction from univariate analysis (HR = 4.87, 95% CI 1.08–21.98, favoring delayed) to multivariable analysis (adjusted HR = 0.267, 95% CI 0.048–1.491). This reversal is a statistical artifact caused by multicollinearity between ruxolitinib timing and HLA matching and must not be interpreted as evidence that immediate initiation improves survival.

In exploratory Fine-Gray models (**Table 3**), immediate initiation was associated with higher NRM (adjusted sHR 4.65, 95% CI 1.47–14.72, P = 0.009). For relapse, the adjusted sHR for immediate versus delayed initiation was 2.17 (95% CI 0.85–5.56, P = 0.095). In the OS model, the reversal of the HR direction for ruxolitinib timing is a well-documented statistical artifact of severe multicollinearity and small sample size and should not be interpreted as any evidence of a survival benefit with immediate initiation. The point estimate for relapse suggests a possible increased risk with immediate initiation, but the P value indicates this result could be due to chance; a larger sample is required for precise estimation.

## 4 Discussion

In this concurrent two-strategy study, delaying ruxolitinib until engraftment was associated with a clinically meaningful reduction in 100-day grade II–IV aGVHD and 2-year NRM, along with superior OS. The unadjusted absolute risk difference was 32.1% for aGVHD and 26.6% for NRM in the full cohort; in the PSM cohort, the corresponding absolute reductions were 33.4% and 25.0%, respectively. The observed lower CMV reactivation and grade ≥ 3 hematologic toxicity in the delayed group are consistent with the biological rationale involving preservation of early JAK-STAT-dependent engraftment and antiviral immunity (14, 15, 20, 23), although the observational nature of this study precludes mechanistic confirmation. PSM analysis confirmed the advantage (grade II–IV aGVHD: 8.3% vs. 41.7%, P = 0.039), supporting the robustness of the findings.

An important consideration in interpreting the full-cohort comparison is the perfect collinearity between haploidentical transplantation and ATG use. The delayed group had both a higher proportion of haploidentical transplants (85.0% vs. 47.4%) and correspondingly higher ATG use. While haploidentical transplantation is associated with increased aGVHD risk, ATG is a potent GVHD prophylactic agent in its own right. The net effect of these opposing forces cannot be determined from this study alone. The lower aGVHD rate in the delayed group, despite higher haploidentical donor proportion, is a noteworthy finding that persisted after PSM—which balanced both HLA matching and ATG use to SMD < 0.1. However, the contribution of ATG to the observed benefit cannot be isolated from that of ruxolitinib timing, and future prospective trials should be designed to disentangle these effects through stratified randomization.

The present study was conducted within the “Beijing Protocol” platform, which uses ATG-based myeloablative conditioning and has been the backbone of haploidentical transplantation in China since its seminal description (17, 18). In the recent era, PTCy-based platforms have emerged as an international standard for GVHD prophylaxis, with accumulating prospective data from large single-center cohorts. Asensi Cantó et al. reported a 180-day cumulative incidence of grade II–IV aGVHD of 21% and grade III–IV of 11% in 385 patients receiving PTCy/sirolimus/MMF after myeloablative allo-HSCT (4), confirming the efficacy of PTCy-based strategies. The same group further characterized cGVHD in 600 consecutive PTCy-based transplants, reporting a 1-year moderate-to-severe cGVHD cumulative incidence of 22%, with only a minority of patients requiring systemic corticosteroids (5). These PTCy-era benchmarks contextualize our aGVHD rates and highlight that our 14-day ruxolitinib strategy, when delayed until engraftment, achieves comparable or superior aGVHD control (10.0% grade II–IV) within a fundamentally different prophylaxis platform.

Regarding relapse risk, the adjusted sHR of 2.17 (95% CI 0.85–5.56, P = 0.095) does not meet the conventional threshold for statistical significance. However, the point estimate suggests a possible increased relapse risk with immediate initiation, and the wide confidence interval—spanning from a protective association to a five-fold increase in risk—precludes a definitive conclusion that relapse risk is equivalent between strategies. The possibility of a clinically meaningful increase in relapse risk with immediate initiation cannot be excluded, given the point estimate in the exploratory multivariable model. Preclinical studies have demonstrated that short-course JAK1/2 inhibition can reduce experimental murine aGVHD while preserving graft-versus-tumor effects (24, 25), providing mechanistic plausibility for the dissociation between GVHD reduction and preserved anti-leukemic activity. The REACH3 3-year final analysis reported low malignancy relapse events during long-term ruxolitinib therapy (8), and the GETH-TC real-world study by Escamilla-Gómez et al. observed no evidence of increased relapse risk in 352 adult and 42 pediatric patients treated with ruxolitinib for steroid-refractory GVHD (9). However, the REACH3 and GETH-TC populations (steroid-refractory cGVHD and steroid-refractory aGVHD/cGVHD, respectively) differ substantially from our prophylactic cohort, and these data should be extrapolated with caution. The possibility of a clinically meaningful difference in relapse risk should be rigorously monitored in future prospective trials. Pre-transplant MRD positivity was associated with inferior OS in the exploratory Cox model (adjusted HR 7.81, P = 0.038), consistent with its established role as a powerful independent prognostic factor across multiple multicenter studies (26–30). Our data demonstrate that even in MRD-positive patients (n = 21 in this cohort), delayed ruxolitinib prophylaxis was associated with reduced GVHD and NRM without detectably attenuating the GVL effect, although the small sample size and wide confidence intervals preclude definitive conclusions regarding the MRD-stratified effect of ruxolitinib timing.

In the exploratory haploidentical subgroup, grade II–IV aGVHD was 11.8% vs. 55.6% (P = 0.028) and 2-year OS rate was 94.1% vs. 55.6% (P = 0.006). In the absence of a statistically significant interaction test, the apparently larger benefit in haploidentical recipients remains a hypothesis-generating observation. The concurrent two-strategy design is a major strength of this study. Unlike sequential cohort designs where treatment strategies change over time, both immediate and delayed ruxolitinib initiation were used simultaneously throughout the study period, minimizing the confounding effects of temporal changes in supportive care, conditioning regimens, and transplant techniques that have limited the interpretability of previous studies (13, 31). The consistent null effect of calendar month across all models reinforces that our results are not driven by temporal trends. This methodological advantage is particularly evident when comparing our design with the randomized phase II trial by Moiseev et al. (11), which used a sequential enrollment design and demonstrated noninferiority of PTCy-ruxolitinib to PTCy-tacrolimus-MMF. Our concurrent design provides complementary evidence that the observed benefit of delayed ruxolitinib initiation is not attributable to period effects. The randomized nature of the Moiseev trial provides superior internal validity for the comparison of PTCy-ruxolitinib versus PTCy-tacrolimus-MMF, while our study specifically addresses the timing question within an ATG-based platform.

The descriptive external comparison with standard prophylaxis is subject to critical biases: non-random assignment due to socioeconomic factors, differential loss to follow-up, and 14 unmatched patients from the standard cohort (suggesting that the matched standard cohort may not be fully representative). The 2-year OS rate of the delayed group was 90.0% in both the internal full cohort and this external comparison; given the substantial biases, this numerical concordance should not be overinterpreted. This comparison is presented solely for descriptive completeness and cannot support clinical inference.

cGVHD cumulative incidences were similar between groups (moderate-severe: 15.0% vs. 15.8%). Observed differences in cGVHD organ involvement patterns are purely descriptive given the extremely small number of events (6 vs. 4 cases). Our study is underpowered for cGVHD endpoints. The similar rates of moderate-severe cGVHD observed between groups, while likely underpowered, are consistent with contemporary reports, reflecting the evolution in cGVHD management over the past decade (32). The cGVHD data from the PTCy era reported by Asensi Cantò et al. (5) provide an informative benchmark: their 1-year moderate-to-severe cGVHD cumulative incidence of 22% with a 27% rate of systemic corticosteroid-free management illustrates the shifting landscape of cGVHD in the modern era.

Our 14-day short-course regimen differs from longer courses used in previous prophylactic studies (10, 22) and from the prolonged maintenance approach (median start day +45, up to 24 cycles) investigated by DeFilipp et al. (12), which achieved a moderate-severe cGVHD rate of 16% at 2 years and a cGVHD requiring systemic therapy rate of 9.5% at 1 year. The similar aGVHD efficacy and lower hematologic toxicity with our 14-day course raise the hypothesis that shorter courses may be sufficient when appropriately timed, although pharmacokinetic confirmation of therapeutic drug levels was not performed in this study. The DeFilipp trial provides compelling evidence that prolonged JAK inhibition effectively suppresses cGVHD, but the optimal timing of initiation for aGVHD prevention—the focus of our study—remained unaddressed. Compared with the peri-transplant initiation approach reported by Kröger et al. (13)—which achieved a 32% grade II–IV aGVHD incidence in myelofibrosis patients, higher than the 10% in our delayed group—our strategy avoids disruption of the peri-engraftment period. Unlike the calcineurin inhibitor replacement strategy investigated by Zhao et al. (19), we integrate short-course ruxolitinib as a time-limited adjunct to the established “Beijing Protocol” backbone (17, 18). This approach aligns with evidence supporting the importance of JAK inhibitor timing, including the demonstration by Abboud et al. that itacitinib can effectively prevent GVHD in haploidentical transplantation when appropriately timed (33), and the review by Abedin and Hamadani (34) which emphasizes the need for refined prophylactic strategies that balance immunosuppression with immune reconstitution.

The difference in CMV reactivation cumulative incidence between groups (35.0% vs. 73.7%) is substantial. However, our interpretation is severely limited by the absence of donor and recipient CMV serostatus data, which are the single most important determinants of post-transplant CMV reactivation. If the immediate group had a higher proportion of high-risk serostatus combinations (e.g., donor-positive/recipient-positive), the observed difference could be partially or entirely attributable to this imbalance rather than to ruxolitinib timing. Biologically, JAK1/2 signaling is a critical mediator of type I (IFN-α/β) and type II (IFN-γ) interferon responses, which activate natural killer cells and cytotoxic T lymphocytes responsible for controlling CMV replication (23). Immediate JAK inhibition on day +1 likely impairs interferon-mediated antiviral immunity during the period of maximal ATG-induced immunosuppression, whereas delaying ruxolitinib until engraftment allows initial reconstitution of innate antiviral effector functions. However, given the missing serostatus data, the CMV reactivation difference can only be described as a phenomenon and cannot be attributed to a drug-timing effect with any degree of confidence. Formal immune reconstitution profiling was also not performed.

Important limitations require explicit acknowledgment, ordered by significance. First, and most critically, donor and recipient CMV serostatus was not available for analysis, substantially limiting the interpretation of CMV reactivation differences. Second, this is a single-center, retrospective study with a small sample size (n = 39) and no formal power calculation, increasing the risk of both type I and type II errors; all multivariable models are severely overfitted (events-per-variable ratio ≈ 2.3–3.0, far below the recommended minimum of 10–20). Third, group assignment was based on clinical judgment rather than randomization, introducing potential selection bias; PSM can only balance measured covariates, and residual unmeasured confounding may persist. Fourth, haploidentical transplantation and ATG use are perfectly collinear in this cohort; their individual effects on outcomes cannot be separated, and the higher ATG exposure in the delayed group may have contributed to the observed benefit independent of ruxolitinib timing. Fifth, immortal time bias is inherent in comparing immediate versus delayed initiation; a landmark analysis at day +14 confirmed consistent results, aided by the fact that all deaths occurred after day 100. Sixth, the external comparison with the standard prophylaxis cohort is subject to significant selection bias; socioeconomic factors may have influenced group assignment, as ruxolitinib was not covered by national health insurance in China during the study period, and lower socioeconomic status is independently associated with inferior transplant outcomes. Seventh, formal immune reconstitution profiling was unavailable. Eighth, subgroup analyses are exploratory and underpowered; no formal interaction tests were performed. Ninth, the “Beijing Protocol” backbone may limit generalizability to PTCy-based platforms (4, 5, 11). Tenth, median follow-up of 42.1 months may be insufficient for late cGVHD and late relapses. Eleventh, the study was not powered for rare outcomes. Twelfth, aGVHD grading was retrospective without prospective blinding. Thirteenth, *post-hoc* study registration precludes confirmation of pre-specified endpoints; secondary and exploratory endpoints in particular may be subject to reporting bias and should be interpreted with caution. Fourteenth, ruxolitinib dosing lacked pharmacokinetic validation; whether the 5 mg twice-daily dose consistently achieved therapeutic drug levels cannot be confirmed. Fifteenth, grade ≥ 3 hematologic toxicity reflects both transplant-related myelosuppression and potential drug effects, compounded by the drug-free interval in the delayed group. Sixteenth, platelet engraftment assessment did not account for the time interval between ruxolitinib initiation and platelet recovery. Seventeenth, no formal adjustment for multiplicity was performed; all P values are nominal. Eighteenth, the distribution of conditioning regimens (BuCy-based vs. TBI-based) was not formally compared between groups, and potential differences in conditioning intensity could have influenced aGVHD risk and hematologic toxicity; however, the small sample size precluded adjustment for this variable. The primary conclusions are based on the consistency of results across both co-primary endpoints and sensitivity analyses rather than on any single P value.

Future trials should incorporate systematic collection of donor and recipient CMV serostatus, serial immune monitoring, biomarker-guided risk stratification (35, 36), optimized ATG dosing based on recipient absolute lymphocyte count (37), pharmacokinetic assessment of ruxolitinib levels, and formal sample size calculations. A multicenter randomized controlled trial with stratification by donor type and pre-transplant MRD status is required to validate these findings before any change in clinical practice can be recommended.

## Conclusion

Despite the aforementioned limitations, these hypothesis-generating results provide a rationale for prioritizing delayed ruxolitinib initiation in future prophylactic trials. In this retrospective, single-center study, delaying short-course ruxolitinib until neutrophil engraftment was associated with reduced aGVHD, NRM, and CMV reactivation, and improved survival after myeloablative allo-HSCT. Although no statistically significant difference in relapse risk was observed between groups, the point estimate from exploratory models suggests a possible increased relapse risk with immediate initiation that requires confirmation in larger studies. These findings warrant confirmation in a multicenter randomized trial with adequate sample size, stratified by donor type and pre-transplant MRD status.

## Data Availability

The original contributions presented in the study are included in the article/supplementary material. Further inquiries can be directed to the corresponding author.
